# Quality of Life as Medicine. II. A Pilot Study of a Five-Day “Quality of Life and Health” Cure for Patients with Alcoholism

**DOI:** 10.1100/tsw.2003.67

**Published:** 2003-09-03

**Authors:** Soren Ventegodt, Joav Merrick, Niels Jorgen Andersen

**Affiliations:** The Quality of Life Research Center, Copenhagen K, Denmark

**Keywords:** Quality of Life, QOL, screening, questionnaires, SEQOL, alcoholism, human development, holistic medicine, public health, Denmark

## Abstract

Alcoholism can be understood as a self-treatment for existential pain. A 5-day treatment was designed to relieve this psychological pain and existential anxiety, and thereby diminish the need for self-treatment with alcohol. The basic principle behind the treatment was holistic, restoring the quality of life (QOL) and relationship with self, which according to the life mission theory happens when life-denying views are corrected and inner emotional conflicts are solved. The method in this treatment was a course with teachings in philosophy of life, psychotherapy, and body therapy. The synergy attained was considerable and the outcome demonstrates that in the course of 1 week, people have time to revise essential life-denying views and to integrate important, unfinished life events involving negative feelings. This was demonstrated by an improved QOL and a decrease in their dependency and need for alcohol abuse. In the week before, after the 5-day course, and again after 1 and 3 months, the 16 participants completed the SEQOL questionnaire on QOL and health. This was a pilot study based on a pre-experimental design, without a control group and without clinical control. Common for the group were a low QOL, numerous health problems, and alcohol dependency in spite of treatment with Antabus® (disulfiram). The study showed an increase in QOL from 57.6% before the course to 69.4% 3 months after the course, or an improvement in QOL of 11.8%. There was a 24.0% improvement in self-perceived mental health, and satisfaction with health in general was improved by 11.1%. The total sum of health symptoms in the group was reduced from 59% of maximum to 33%. It is concluded that for this small and motivated group with alcohol problems, it was possible to improve QOL and health in only 5 days with a holistic treatment that combined philosophy of life, psychotherapy, and body therapy, but the results are not final. Further research is needed.

## INTRODUCTION

Alcoholism is a public health issue costing every modern society billions of dollars in direct and indirect expenses[1,2]. The aetiology of alcoholism is not well understood. Some researchers have found a genetic connection, while others have suggested that alcoholism is basically self-treatment for anxiety and psychological pain[3,4]. This is in accordance with the known tranquillising effect of alcohol.

The most famous medical cure for alcoholism is treatment with disulfiram (Antabus®) and recently two more drugs, acamprosate and naltrexone, have been introduced to treat severe alcohol dependence and reduce alcohol intake[5]. The need of drinking seems not to be removed by this treatment, but can be helped by a psychosocial intervention, the most famous of these being the Minnesota 12-Step approach[6,7]. The Minnesota model is based on admitting the alcohol dependency to peers and in basic trust. It starts with a 5-week in-patient course, helping people to realise their poor situation and their need for personal improvement. The Minnesota Model demonstrates that psychosocial treatment can be very effective, but many alcoholics hesitate to participate, as they feel uneasy with the religious concept, it is also fairly expensive (in Denmark about 8,000 USD for the first 5 weeks), and many alcoholics are not helped by this intervention in the long term.

Since 1990, the Quality of Life Research Centre in Copenhagen has studied the connection between quality of life (QOL) and a large number of factors, one among them the abuse of alcohol[8,9]. In our work with QOL, we focused on measuring global and generic QOL, using SEQOL (a self-administered, theory-based questionnaire[10]) and, through the Copenhagen Perinatal Birth Cohort 1959–61, looked at long-term aspects of QOL compared with events in pregnancy and early childhood[9,11].

We believe there is a connection between QOL and health in the way that the connection is hypothesised to be causal from QOL to health[12], meaning that health improves when QOL improves. We also believe that this hypothesis (that QOL and self-perceived health are susceptible dimensions that can be improved considerably in a short time) must be scientifically tested through intervention studies. The factors that were subject to influence were brought together in a concept for the intensive 5-day QOL and health course (5D-QOL intervention). The purpose of the experiment was to test qualitatively and quantitatively the hypothesis that QOL and subsequently subjective health can be improved effectively by a combination of philosophy of life, psychotherapy, and body therapy, supporting the salutogenetic process[13,14].

## THE PHILOSOPHY BEHIND THE STUDY

According to the life mission theory[12], global QOL is improved when the patient lets go of negative beliefs. Negative beliefs according to the theory are based on emotional pain, which are “deposited” in body and mind, giving poor QOL, poor mental and physical health, with a low ability of functioning. The philosophy behind this study is somewhat simplified for practical reasons. The human being is here described as existing in three different inner worlds, which are parallel to each other: a mental world, an emotional world, and a physical world. The reason for compartmentalising human existence into these three worlds (the mind, feelings, and the body) is that they seem to live their own lives inside us to such a degree that they are normally taken as free to develop independently of each other. Training and teaching (for example, in philosophy)

can improve our understanding of life. When processing our personal history, therapy can help us towards a healthier emotional life. When we work with our body, we become more present in it, less shameful about our sexuality for example, and we can come to feel that “the energies flow”. Such subjective experiences seem to be highly important for the cure for alcoholism. But most important is the experience of healing one’s existence, of finally being oneself: the person you were meant to be[12]. It seems that combining work with the body, the feelings, and the mind can give the patient this unique and important experience, which often gives the patients a permanent betterment. This seems to explain the observed large synergy of these three methods. It is important to stress that the effect could also follow from other factors, like a lucky presentation for the subject in the study, rendering them highly motivated for personal development.

Our approach differs from the normal approach in that we attempt to solve the inner conflicts that we believe are the real cause of alcoholism. In the Alcoholics Anonymous program, the participant must accept that the urge for alcohol is a permanent feature of the personality, and that the person is a “dry alcoholic” after successfully completing treatment. With our holistic approach, we hope to help the patient not to be an alcoholic anymore, but to be a whole, happy, and free person.

The 5-day treatment consisted of the following three elements and their axiomatic fundamentals.

## Philosophy of Life: Find Your Constructive Philosophy of Life

1. **Reorientation towards life.** The good life is a life where we realise ourselves by knowing ourselves and our own wants, needs, resources, and potentials, and by knowing the possibilities in the world and the surroundings and using these to the full. Our hidden resources are acknowledged. The meaning of life is to make all our relationships good and fruitful — to create value for ourselves and our surrounding world.
2. **Identification of bad attitudes.** The problem we are struggling with is ourselves or, more precisely, all the bad attitudes we have acquired through our personal history of defeats and victories, confirmations and invalidations.
3. **Identification of inappropriate behaviour, which makes life what it is.** We make our own life, but usually do not see it. Our consciousness is self-affirmative, which means that what we believe we always make happen in our reality, whereupon we are confirmed in what we believe.
4. **Identification of better attitudes, which lead to a better life.** Life contains wisdom, joy, and vital energy, which can be acknowledged and expressed in a new belief in life and its possibilities, especially our own influence on our own existence.
5. **Identification of the new, constructive behaviour.** Better behaviour matches the new and better attitudes and forms life as better, more healthy, and positive.
6. **Decisions and consequent action on the new, better attitudes.** When we commit ourselves to practise better attitudes to life in our everyday lives, our lives change. Negative aspects are cleared from our personalities and we find a new freedom to flourish and develop.

## Psychotherapy

1. **The good life is about feeling good by creating and maintaining our own inner emotional space.** In this space we build up our being, our self-esteem, our confidence, and our happiness.

2. **A prerequisite for this is to know and respect the limits of our emotional space.** The art of living means defining our own limits both wisely and correctly — to delimit ourselves and see ourselves as separate from the surrounding world and other people, to simultaneously experience ourselves as being a harmonious part of the whole.
3. **The difficulties arise, because for years — our personal history — we have carried a lot of bad attitudes and correspondingly inappropriate behaviours** around with us, which cause us to violate our own space and our own limits and allow others to offend us. When we let go of all the negative aspects we have collected through life from unfinished events in our personal history, life again has room to blossom within us.
4. **Life with others is first and foremost about appreciation and honest and respectful communication.** This presupposes that we have our own inner space and limits in place.
5. **With the ability to act without violating the space and limits of ourselves and others and with the ability to communicate honestly and respectfully, we can rebuild our lives.** We can become free, living human beings who accept and trust other people without using force, i.e., without demanding, criticising, and controlling each other. The ability to communicate can improve gradually to encompass bigger and bigger parts of our surroundings and our personal universe.

### Body Therapy

1. **All suffering is due to blockages** of the body's "vital energy", which again is connected to our lies and denials of life. When we let go of our physical tensions, we release body energy and feelings.
2. **When we touch areas on our body, we can easily feel the blockages**, and when we work with ourselves, we can also discover from where in our personal history these blockages stem and the painful feelings and vital denials they are connected to.
3. **QOL in the sense of physical well being is about being present in all parts of the body.** It is necessary to recapture the inner space of the body. Your breathing releases the old blockages and sets free the feelings that have been tied up in the body for a long time. A blockage is a regional withholding of breath and thus a withdrawal of our attention from the area.
4. **Body parts are connected in a subtle system of "body energy": biological information, awareness, and purpose of life.** When we have acknowledged this system intuitively, it is simple to move problems from the physical side to the psychological side. To put it another way, you can transfer the pain from being physical to being psychological after which you can confront it and get rid of it for good.
5. **"Body energies" appear as emotional interpretations of the "stream of information" in the body.** The body possesses a perfect knowledge of why it became ill and how to become well again. Therefore, the art of becoming well again is totally about learning to listen to the body.

## METHODS

The study was an intervention study without a control group. It was not an experiment, but a study based on a pre-experimental design. The group of participants in the course was comprised of alcoholics on disulfiram or Antabus® with many years history of alcohol abuse. Common for the group was poor QOL and many health problems.

The end points or dependant variables of the study were QOL, self-perceived mental health, and satisfaction with one's health and the number and intensity of the health problems.

A total of 18 long-term alcoholics from the town of Elsinore in the north of Copenhagen, who went to control at an alcohol clinic in Frederiksborg County, were enrolled in the course and 16 of those completed the course. The primary contact was through a lecture on personal development given at the institution on “How to improve your QOL”. The participants volunteered for the experiment after they had been introduced to the concept and the underlying philosophy of life, whose main message was that all people possess large hidden resources to be used for improving global QOL and health.

The participants were measured the week before and after the 5-day intervention course, and again after 1 and 3 months (response rate 17/18, 14/18, 14/18, and 12/18, respectively). The self-administered “Questionnaire on Quality-of-Life and Health for Alcoholics” was used. It contains the SEQOL questionnaire for self-evaluation of QOL and health[8,10]. The first-mentioned questionnaire was developed for the Danish “Quality-of-Life” Population Survey at the University Hospital of Copenhagen[8,10]. The questionnaire contained 317 questions. A qualitative form containing open questions and textual responses was also used and the forms were completed in the usual surroundings of the participants.

The group was split into two smaller groups that received therapy simultaneously. Mornings and afternoons were devoted to lectures on philosophy of life to sum up the events of the day and put them into perspective.

Evenings were for individual sessions in psychotherapy and body therapy for those especially in need of this. No strangers, observers, or friends were allowed into the therapy rooms, while the philosophy room was videotaped for documentation. All participants were bound to secrecy regarding the experiences of others. The course took place at the education center “LO Skolen” in Elsinore, Denmark.

The following concept for measuring global, generic QOL was used[8]:

1. A clear definition of the QOL
2. A philosophy of life on which the definition of QOL was based
3. A theory that makes this philosophy operational by deducing questions that are unambiguous, mutually exclusive, and comprehensive as a whole and establishing the relative weights of each question
4. A number of response options that can be quantitatively interpreted on a fraction scale
5. Technical quality in terms of reproducibility, sensitivity, and well-scaledness (appropriate scale characteristics)
6. The survey must be meaningful to researchers, respondents, and those who use the results (including criterion validity)
7. An appreciation of the aesthetic dimension

## RESULTS

Results (see Tables 1 and 2, and Fig. 1) are from the four measurements of QOL, health, and dependency of alcohol (sum of symptoms) of the 18 participants with the SEQOL questionnaire[8,10]. Immediate subjective well being was measured with only one question on a five-point symmetric Likert scale, as was satisfaction with life and happiness. Satisfaction of needs was measured with five questions according to a modified theory of needs based on Maslow’s hierarchy of needs. All five questions were rated on five-point Likert scales. The composite global QOL measure “family, work, and leisure time” rated global QOL at home, at work, and in the leisure time using three questions and three five-point Likert scales. The “quality of relationships” was given an average of the rating of all close relationships on five-point Likert scales. Total QOL was calculated as usual in SEQOL according to the integrated QOL theory (IQOL theory), except for the objective factors not included in this study [see 8].

**TABLE 1**
**Measurings of Global QOL and Self-Assessed Health with the SEQOL Questionnaire, Before and After the 5-Day QOL Intervention**

| | T1 | T2 | T3 | T4 | T4-T1 | *p* Value |
|---|---|---|---|---|---|---|
| Immediate subjective well being | 56.7 | 70.0 | 75.3 | 78.3 | 21.6 | < 0.05 |
| Satisfaction with life | 60.0 | 68.6 | 70.0 | 75.0 | 25.0 | < 0.05 |
| Happiness | 56.7 | 64.3 | 64.7 | 68.3 | 11.6 | ns |
| Satisfaction of needs | 63.2 | 68.6 | 67.1 | 75.0 | 11.8 | < 0.05 |
| Family, work, and leisure time | 65.6 | 71.4 | 73.3 | 72.4 | 6.8 | ns |
| Quality of relationships | 60.0 | 65.4 | 69.1 | 68.5 | 8.5 | < 0.05 |
| **Total QOL** | **57.6** | **64.6** | **66.4** | **69.4** | **11.8** | **< 0.05** |
| Self-assessed physical health | 76.7 | 71.4 | 77.1 | 78.3 | 1.6 | ns |
| Self-assessed psychological health | 50.0 | 65.0 | 71.4 | 74.0 | 24.0 | < 0.05 |
| Satisfaction with health | 62.2 | 65.7 | 71.3 | 73.3 | 11.1 | < 0.05 |
| Sum of health problems | 5.9 | 5.4 | 3.7 | 3.3 | 2.6 | < 0.05 |
| **Total self-assessed health** | **63.9** | **67.9** | **72.0** | **76.7** | **12.8** | **< 0.05** |

T1: the week before the course; T2: the week after; T3: 1 month after; T4: 3 months after. The number (N) of participants answering the questionnaire: N = 18 at T1, N = 14 at T2, N = 15 at T3, and N = 12 at T4. The level of significance for the QOL measures is estimated using Table 9.1 in [8]. ns: nonsignificant.

**TABLE 2**
**Health Problems, Relieved by the 5-Day QOL Intervention, Measured by the SEQOL Questionnaire**

| | Measured Before Intervention | Measured After Intervention | *p* Value | N |
|---|---|---|---|---|
| Pain or discomfort in back or buttocks | 1.93 | 1.67 | 0.04 | 15 |
| Headache | 1.67 | 1.40 | 0.04 | 15 |
| A rapid heart beat | 1.33 | 1.06 | 0.04 | 15 |
| Difficulty sleeping (insomnia) | 1.80 | 1.27 | 0.001 | 15 |
| Impairment of memory | 1.80 | 1.27 | 0.01 | 15 |
| Melancholy, depression, or unhappiness | 1.53 | 1.00 | 0.006 | 15 |
| Indigestion, diarrhoea, or constipation | 1.47 | 1.00 | 0.01 | 15 |
| Pain (% of maximum, all measured pains) | 37.8% | 26.7% | 0.005 | 15 |
| Sum of health problems | 5.9 | 3.3 | 0.001 | 14 |

Health problems not found to be significantly relieved in this study were: stress; uneasiness, nervousness, restlessness, or anxiety; eczema, rash, or itching; cold, head cold, or cough; difficulty in breathing or breathlessness. When all pains tested by the questionnaire were summed up to one measure of global chronic pain, it appears that the pain level of the participants is relieved by the 5-day QOL intervention, similar to our earlier finding that the intervention is efficient in relieving chronic pain[15]. In this study we found a 29.4% reduction in pain; 44.1% of all health problems disappeared from T1 to T4 as measured by SEQOL. Level of significance is calculated using the TTEST procedure in SAS.

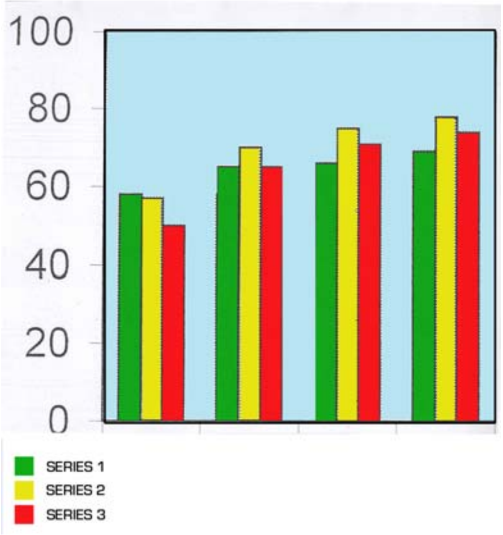

**FIGURE 1.** Immediate subjective well being (yellow), total QOL (green), and psychological health (red) before and 1 week, 1 month, and 3 months after the 5-day intervention as measured by the SEQOL questionnaire. It is interesting that the participants seem to continue to improve their QOL and health in the months after the intervention. We believe that this is because they are using the tools and concepts for personal development, which they are trained to use during the 5-day course.

Self-assessed physical health, self-assessed psychological health, and satisfaction with health were all measured with one question using a five-point Likert Scale. "Sum of health problems", was a measure of the total amount of health problems and self-assessed on a three-point Likert scale, where 1 means no symptoms at all, 2 means that the respondent had the health problem to a small extent, and 3 means that the respondent suffered severely from the health problem. "Total self-assessed health" was calculated as a weighted average of the four health measures as described by Ventegodt[8]. The psychometric property of the used five-point Likert scale in global QOL instruments was documented in other papers[10,15].

## Quality of Life

At the first measurement, the QOL of the participants was clearly below average. The subjective dimensions of immediate subjective well being, satisfaction with life, and happiness rated 56.7, 60.0, and 56.7%, respectively, as compared to normally about 70.0% for these dimensions. This group was really low in QOL.

Compared to this, QOL at the second measurement was considerably higher and a little below the normal level. Immediate subjective well being, satisfaction with life, and happiness rated now 70.0, 68.6, and 64.3%, respectively. The improvement was immediate and surprisingly large.

We expected the values to slowly return to normal, but we found at the third and fourth measuring that QOL was still rising. The third QOL measuring showed that immediate subjective well being, satisfaction with life, and happiness rated 75.3, 70.0, and 64.7%, respectively. The fourth QOL measuring showed that immediate subjective well being, satisfaction with life, and happiness continued upwards to almost incredible values of 78.3, 75.0, and 68.3%, respectively. It must be admitted that we lost five of the participants during this period so that response rate fell to 66.6%, but this is still fair even if it can explain some of the difference. At the end of the study, the participants could not be discriminated from the normal population by their global QOL ratings.

## Health

Data on health problems were collected by means of a questionnaire included in SEQOL with some questions originally developed by the Danish Institute for Clinical Epidemiology.

Before the QOL course, the first measurement showed a considerable difference between the 18 participants and the general population with regard to health. The self-assessed physical and mental health rated 76.7 and 50.0%, respectively, the first being normal, the second far below population average of 71.0%, but equal to that of mental health patients. Satisfaction with health rated 62.2% compared to an average of the general population of 72.0%. Another important health dimension was the number and intensity of symptoms from all the organ systems of the organism. We included a screening of this, which we called the sum of symptoms. The group rates 59.0% of maximum before the QOL intervention. This was a very negative result and far below the average of the general population.

The calculated "total health" based on these data was assessed to 63.9% compared to the general level of 71.0% in the normal population, a difference of 9.8 and 7.1%, respectively. The most important observation here seems to be the self-assessed mental health, in accordance with the hypothesis that psychological pain and discomfort was the real, but hidden reason for the dependency of alcohol.

Immediately after the 5-day QOL course, the second measurement showed a considerable improvement in self-assessed mental health (while the physical health went down). The self-assessed physical and mental health now rated 71.4 and 65.0%, respectively, both now being within the normal range. Satisfaction with health rated 65.7% coming close to the general population of 72.0%. Sum of symptoms now rated to 54.0% of maximum, before the QOL intervention, still rather bad and below the average. The calculated "total health" was now assessed to 67.9%, also close to normal.

As was the case with the QOL, health also continued to improve in the month after the course. One month after the 5-day QOL course, the third measurement again showed a considerable improvement in self-assessed mental health now rating 71.4%. The physical health had now risen to 77.1%. Satisfaction with health rated 71.3%, identical to the rating of the general population of 72.0%. Sum of symptoms rated 37.0% of maximum in the third measurement, a remarkable improvement. The calculated "total health" was now assessed to 72.0%, which was normal.

The fourth measuring showed that self-assessed mental health rated 74.0%. The physical health rated 78.3%. Satisfaction with health rated 73.3%. Sum of symptoms rated 33.0% of maximum, a surprising development. The calculated "total health" was now assessed to 76.7%.

We had some scepticism about the results and therefore make a conservative calculation of our results. Even if we took an average of the two first measurings as basis and compared this value with the two later measurings, the improvement was both statistically significant and remarkably large (see Table 1).

A very interesting observation was that the holistic cure seemed to alleviate symptoms from all organ systems, like upper and lower back pain, depression and unhappiness, indigestion, palpitations, impairment of memory, insomnia, and headaches (see Table 2). Pain in general was reduced by 29.4% in accordance with earlier findings that the 5-day QOL cure is efficient in relieving chronic pain[16].

## DISCUSSION

We were genuinely surprised that quality of life (QOL) and health, especially self-assessed mental health, seemed to continue improving in the months after the 5-day QOL-course. We believe this is primarily a consequence of the philosophy of life that stresses personal development.

The size of the changes in QOL, health, and alcohol dependency were generally unexpectedly large. Considering that only a 5-day course of intervention took place, the difference of 10 to 20% in the above-mentioned dimensions from before until after the course must be seen as great and distinctive. We were quite happy with the therapeutic results after the course, but had a conservative expectancy to the outcome over time as it is normally believed that life cannot be changed in a few days, but that the QOL only temporary can be improved.

The fact that the gains of the participants were still rising 3 months later is promising, but still it is not likely that they become permanent without reinforcement, although it seemed fair to expect that the measured QOL and health dimensions will not return to their previous low level. Good attitudes are known to decay over time and must be reinforced constantly, something that is part of human nature. The new understanding of life and understanding of the basic principles of personal development, which the course provided to the participants, were thought to be more permanent.

It must be considered a possibility that we collected patients that were highly motivated for personal development, so that the participants are different from the average alcoholic. The area from which we recruited the participants (the North part of Sealand) is known to have a higher socioeconomic level than the rest of Denmark and it is possible that the participants were more highly motivated and more resourceful than the average Danish alcoholic.

The scientific method suffered from the lack of a control group, because it is easy to suspect that just being removed from your everyday routine and placed together with others in a beautiful conference centre next to the sea for 5 days and nights will make a great momentary difference to your QOL.

The group was well known as chronically, mental ill from disulfiram- or Antabus®-resistant alcohol dependency and we believe that the treatment group was typical for the heavy segment of the Danish alcoholics, but they might be more motivated than average to help themselves, because of the way they were initially recruited (by a lecture recommending intensive, personal development). At the beginning of the course the participants were socially poor functioning and none of the participants were able to work. We made a 2-day follow-up for the group after 6 months and were informed that 3 of the participants had gotten a job 6 months after intervention, and that 9 of the 16 participants, who did complete the course, were feeling a lot better than before the course. Most of the participants continued drinking, but they noted that their drinking patterns had changed from weeks of uncontrolled abuse to just a few days with much more control.

This pilot study looks promising, but no final conclusions can be made, since it is important to repeat such a study under clinically controlled conditions with a larger number of participants and a longer follow-up.

## CONCLUSIONS

Our pilot study showed apparently that a 5-day QOL course combining training in philosophy of life, psychotherapy, and body therapy can give an alcohol abusive person a large, fast, and efficient improvement in QOL and health, and reduce alcohol dependency. This is in support of the hypothesis that people drink because of psychological and existential pain and discomfort. It is not known whether these changes will be permanent or if these promising results can be reproduced in a controlled clinical trial with more participants. Further research in this regard with control groups should be conducted. We hope to be able to follow the Cochrane standard in future "QOL as medicine" studies. We also hope that the international medical community will find this new approach interesting and join us for future cooperation. We hope and encourage that the powerful medical institutions of this world, like university hospitals, private foundations, sponsors, and governments will support this promising line of holistic medicine and psychosomatic research.

## ACKNOWLEDGEMENTS

The intervention has come about through a cooperation between the Quality of Life Research Centre in Copenhagen, Denmark and the Association of Alcoholics, called "Lænken" (The Chain) in Elsinore.

The following foundations have supported the “Quality of Life Survey”: Apoteker-fonden af 1991, De praktiserende lægers Goodwill-fond, JL Fondet, Direktør E. Danielsen og Hustrus Fond, Emmerick Meyers legat, Frimodt-Heineken fonden, Familien Hede Nielsens Fond, Petrus Andersens Fond, Grosserer C.P. Frederiksens Studielegat for medicinske studenter og unge læger and Else & Mogens Wedell-Wedellsborgs Fond, IMK Almene Fond, and others.

The study “Quality-of-Life and Causes for Illness” and “Quality-of-Life and Causes for Illness II: Intervention” with Søren Ventegodt as project leader is approved by the Scientific Ethical Committees for Copenhagen and Frederiksberg under the numbers V100.1762/90 and 01-502/93, as well as supervised by the Danish Data Protection Agency, “Registertilsynet”. The present pilot study was the first part of a psychosomatic research project “Quality-of-Life and Causes for Illness II: Intervention”.

## References

1. Nalpas, B., Combescure, C., Pierre, B., Ledent, T., Gillet, C., Playoust, D., Danel, T., Bozonnat, M.C., Martin, S., Balmes, J.L., and Daures, J.P. (2003) Financial cost of alcoholism treatment programs: a longitudinal and comparative evaluation among four specialized centers. *Alcohol Clin. Exp. Res.* **27(1)**, 51–56.
2. Goetzel, R.Z., Hawkins, K., Ozminkowski, R.J., and Wang, S. (2003) The health and productivity cost burden of the “top ten” physical and mental health conditions affecting six large US employers in 1999. *J. Occup. Environ. Med.* **45(1)**, 5–14.
3. Konishi, T., Calvillo, M., Leng, A.S., Feng, J., Lee, T., Lee, H., Smith, J.L., Sial, S.H., Berman, S.H., French, S., Eysellein, V., Lin, K.M., and Wan, Y.J. (2003) The ADH3*2 and CYP2E1c2 alleles increase the risk for alcoholism in Mexican American Men. *Exp. Mol. Pathol.* **74(2)**, 183–189.
4. Tyndale, R.F. (2003) Genetics of alcohol and tobacco use in humans. *Ann. Med.* **35(2)**, 94–121.
5. Graham, R., Wodak, A.D., and Whelan, G. (2002) New pharmacotherapies for alcohol dependence. *Med. J. Aust.* **177**, 103–107.
6. Anderson, D.J., McGovern, J.P., and DuPont, R.L. (1999) The origins of the Minnesota model of addiction treatment. A first person account. *J. Addict. Dis.* **18(1)**, 107–114.
7. Winters, K.C., Stinchfield, R.D., Opland, E., Weller, C., and Latimer, W.W. (2000) The effectiveness of the Minnesota Model approach in the treatment of adolescent drug abusers. *Addiction* **95(4)**, 601–612.
8. Ventegodt, S. (1996) *Measuring the Quality of Life. From Theory to Practice*. Forskningscentrets Forlag, Copenhagen.
9. Ventegodt, S. (1995) *The Quality of Life and Factors in Pregnancy, Birth and Infancy. Correlation between Quality of Life of 4500 31-33-year-olds and Data about their Parents, Birth and Infancy.* Forskningscentrets Forlag, Copenhagen.
10. Ventegodt, S., Henneberg, E.W., Merrick, J., and Lindholt, J.S. (2003) Validation of two global and generic quality of life questionnaires for population screening: SCREENQOL and SEQOL. *TheScientificWorldJOURNAL* **3,** 412–421.
11. Ventegodt, S. (1999) A prospective study on quality of life and traumatic events in early life — a 30-year follow-up. *Child Care Health Dev.* **25(3),** 213–221.
12. Ventegodt, S. (2003) The life mission theory: a theory for a consciousness-based medicine. *Int. J. Adolesc. Med. Health* **15(1)**, 89–91.
13. Antonovsky, A. (1987) *Unravelling the Mystery of Health: How People Manage Stress and Stay Well.* Jossey-Bass, San Francisco.
14. Antonovsky, A. (1985) *Health, Stress and Coping*. Jossey-Bass, London.

15. Lindholt, J.S., Ventegodt, S., and Henneberg, E.W. (2002) Development and validation of QoL5 for clinical databases. A short, global and generic questionnaire nased on an integrated theory of the quality of life. *Eur. J. Surg.* **168,** 107−103.
16. Ventegodt, S., Merrick, J., and Andersen, N.J. (2003) Quality of life as medicine. A pilot study of patients with chronic illness and pain. *TheScientificWorldJOURNAL* **3**, 520–532.

## BIOSKETCH

**Søren Ventegodt, MD,** is the Director of the Quality of Life Research Center in Copenhagen, Denmark. He is also responsible for a Research Clinic for Holistic Medicine in Copenhagen and is a popular speaker throughout Scandinavia. He has published numerous scientific or popular articles and a number of books on holistic medicine, quality of life, and quality of working life. His most important scientific contributions are the comprehensive SEQOL questionnaire, the very short QoL5 questionnaire, the integrated QOL theory, the holistic process theory, the life mission theory, and the Danish Quality of Life Research Survey, 1991–94 in cooperation with the University Hospital of Copenhagen and the late pediatric professor Bengt Zachau-Christiansen. E-mail: ventegodt@livskvalitet.org. Website: www.livskvalitet.org/

**Joav Merrick, MD, DMSc,** is Professor of Child Health and Human Development affiliated with the Zusman Child Development Center and Division of Community Health at the Ben Gurion University, Beer-Sheva, Israel and presently the Medical Director of the Division for Mental Retardation, Ministry of Social Affairs, Jerusalem and the Director of the National Institute of Child Health and Human Development. He has numerous publications in the field of child and human development, rehabilitation, intellectual disability, disability, health, welfare, abuse, advocacy and prevention. Dr. Merrick received the Peter Sabroe Child Award for outstanding work on behalf of Danish Children in 1985 and the International LEGO-Prize ("The Children's Nobel Prize") for an extraordinary contribution towards improvement in child welfare and well being in 1987. E-mail: jmerrick@internet-zahav.net. Website: www.nichd-israel.com

**Niels Jørgen Andersen, MSc,** Professor, Department of Innovation and Economic Organization, Norwegian School of Management. This department conducts research and provides teaching in central topics related to innovation, business development, management of global companies, business history, and economic organization. Research activities within the Department are related to four core subjects within the discipline: business history, cooperative organizations, business development and entrepreneurship, and finally studies of industries with a special focus on the electricity industry. He is also the dynamic chairman of the nonprofit organisation Stiftelsen Holistisk Medisin Scandinavia, which aims to support the scientific development, research, and documentation of complementary and holistic medicine in Scandinavia. E-mail: niels.j.andersen@bi.no. Website: www.bi.no/users/fgl93013/
